# Gape‐limited invasive predator frequently kills avian prey that are too large to swallow

**DOI:** 10.1002/ece3.11598

**Published:** 2024-07-25

**Authors:** Martin Kastner, Scott M. Goetz, Kayla M. Baker, Shane R. Siers, Eben H. Paxton, Melia G. Nafus, Haldre S. Rogers

**Affiliations:** ^1^ Department of Fish and Wildlife Conservation Virginia Tech Blacksburg Virginia USA; ^2^ U.S. Geological Survey Pacific Island Ecosystems Research Center Hawai'i National Park Volcano Hawai'i USA; ^3^ United Stated Department of Agriculture National Wildlife Research Center Barrigada Guam

**Keywords:** body size, Brown Treesnake, foraging theory, gape limitation, predation success

## Abstract

Gape‐limited predators (e.g., snakes, many fish) are not generally expected to pose a predation threat to prey that are too large for them to swallow. However, the extent to which snakes predate on prey that exceed their gape limitation remains largely unknown. We conducted the first study to investigate the influence of both prey and predator sizes on the frequency of ingestion success by snakes in a natural system. We combined survival monitoring of an avian prey species (*Aplonis opaca*) via radio‐telemetry with a survey of the size distribution of their major predator (*Boiga irregularis*) on Guam. This allowed us to assess (1) the frequency of unsuccessful ingestion by the predator, (2) whether the size of the prey predicts ingestion success, (3) whether the size of the predator predicts ingestion success, and (4) the relationship between prey and predator sizes in successful ingestion attempts. We found that nearly half (47.95%) of ingestion attempts by snakes on fledgling birds were unsuccessful, and no instances where unsuccessful ingestion caused the mortality of the snake. Attempts to consume smaller fledglings were as likely to be unsuccessful as attempts to swallow larger fledglings. However, snakes that successfully ingested fledglings were among the largest snakes in the population, and larger than average conspecifics attracted to endothermic prey. The smallest snakes that successfully ingested fledglings attained remarkably high relative prey mass values for their species, consuming prey weighing up to 79.9% of their own mass. Our study indicates that *B. irregularis* routinely predate prey that are too large for them to successfully ingest, which causes mortality to the prey but poses little risk to the predator. The potential reward for snakes in consuming oversized prey may outweigh the inherent risks, while instances of predation that do not result in consumption may have considerable impacts on prey populations.

## Graphical abstract

Gape‐limited predators (e.g. snakes, many fish) are not generally expected to pose a predation threat to prey that are too large for them to swallow, but the extent to which snakes predate on prey that exceed their gape limitation remains largely unknown. We found that nearly half (47.95%) of ingestion attempts by snakes on fledgling birds were unsuccessful, and no instances where sliming unsuccessful ingestion caused the mortality of the snake. The potential reward for snakes in consuming oversized prey may outweigh the inherent risks, while instances of predation that do not result in consumption may have significant impacts on prey populations.

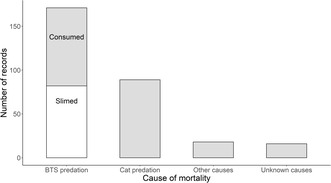

## 1 INTRODUCTION

Predation attempts have the potential to shape the abundance, demographic structure, and behavior of prey populations via both direct and indirect pathways. Broadly, unsuccessful predation attempts are expected to compose the majority of predator–prey interactions, although success rates are highly variable across predator taxa (Vermeij, 1982). For example, most studies on raptorial birds have documented post‐detection predation success rates lower than 20%, while predation efficiency approaching 100% has been reported for crabs, fish, and other taxa, albeit on the most vulnerable life stages of their prey (Abrams, 1989; Vermeij, 1982). Numerous factors relating to prey influence predation success, including behavioral factors like habitat selection (Sih, 1985), evasive behaviors (Christensen, 1996), and learned recognition of predator cues (Acquistapace et al., 2003), as well as physical factors including crypsis (Ruxton et al., 2019), group size and composition (De Luna et al., 2010), health status (Semlitsch, 1990), mechanical defense (Sawyer et al., 2009), and, crucially, the size of prey relative to predators (Weitz & Levin, 2006).

Predators that increase in size as they mature generally broaden their diet by gradually increasing the maximum size of their prey (Christensen, 1996; Vézina, 1985). Indeed, optimal foraging theory predicts that predators should select prey that provide the highest energy return per unit of handling time (Stephens & Krebs, 1986). Conversely, an increase in prey body size relative to the size of a given predator has been shown to greatly reduce predation risk, due either to a reduction in capture or handling efficiency by the predator or their more rapid satiation (Semlitsch, 1990). For example, in New Zealand, juvenile North Island Brown Kiwi (*Apteryx mantelli*) suffer intense predation by invasive stoats (*Mustela erminea*) for their first months of life until they attain a size refuge, beyond which they are no longer vulnerable to stoat predation (McLennan et al., 2004). For gape‐limited predators, such as many fish, lizards, and birds, and most snakes, the upper threshold in prey size is predicted to be dependent on the relationship between the size of the predator's mouth and the widest point of the prey item. The individual‐level risk of predation by a gape‐limited predator is expected to drop abruptly once the widest point of the prey's body exceeds a corresponding measure of the predator's mouth size (Christensen, 1996). However, the degree to which individual predators are capable of judging their own gape size and the consequences of attempting to ingest prey that are too large to swallow are relatively poorly characterized.

Snakes are an almost universally gape‐limited clade that have evolved a variety of behavioral, physiological, and mechanical strategies that enable them to consume larger prey items (i.e., heavier and/or bulkier, sensu Greene & Wiseman, 2023). Snakes serve as a model system for studying how the relative size of predators and prey affects diet and feeding ecology (Greene, 1983; Jayne et al., 2022). Body size in many snake species is positively correlated with the maximum size of their prey (Arnold, 1993; King, 2002; Shine, 1991). However, data on the success of predation attempts in snakes, and its relationship to prey size, remain scarce because of the cryptic nature of many species. Data on snake diet are generally based on gut contents of museum specimens, or prey items that are forcibly regurgitated by free‐ranging snakes, thus only recording successful ingestion events but not reflecting a comprehensive picture of predation attempts (Costa & Trevelin, 2020; Glaudas et al., 2017). Studies on snake predation success often take place in a laboratory setting, which may not be representative of natural conditions for predator or prey (Clark, 2006). Anecdotal reports of unsuccessful predation from nature indicate that success rates may be lower than previously assumed (Costa & Trevelin, 2020). Field‐based empirical studies have found that the success rate of predatory strikes by ambush‐hunting snakes in hitting their targets is generally around 50% or lower (e.g., Clark, 2006; Clark et al., 2012; Glaudas et al., 2017). However, for a gape‐limited predator such as a snake, incapacitating a prey item is only half the battle—they must still manage to swallow it whole.

Results from the few studies that have documented the ingestion success of snakes suggest that they may regularly kill prey that are too large for them to successfully ingest. For example, Shedao Island Pit Vipers (*Gloydius shedaoensis*) are often observed capturing and attempting to feed on birds they are unable to swallow (Shine & Sun, 2003). A recent telemetry study on the survival of koala's (*Phascolarctos cinereus*) found that only slightly more than a third (38%) of the koalas killed by carpet pythons (*Morelia spilota*) were successfully ingested by the snakes (Robbins et al., 2019). There are also many anecdotal reports of snakes attempting to ingest oversized prey (Kornilev et al., 2023). These reports raise the questions of how frequently snakes are unsuccessful in consuming captured prey, and how ingestion success relates to the relative sizes of prey and predators. Aside from energetic reward, metabolic, time, and, in some cases, venom costs, snakes also incur a risk of injury or death when attempting to consume oversized prey (Kornilev et al., 2023). There are therefore competing hypotheses as to why snakes may attempt to ingest such prey: these attempts may either be dangerous mistakes triggered by visual or chemical cues, or alternatively, they are calculated risks where the potential benefits outweigh the likely harm. Sazima and Martins (1990) speculate that predation attempts on oversized prey may be especially prevalent in young snakes, perhaps due to inexperience, or the unavailability of appropriately sized prey. However, Feder and Arnold (1982) predicted that it would be beneficial for snakes to attack large or difficult prey, even if such prey are ingested only occasionally, because of the potential for a substantial energetic reward. This implies that snakes may regularly attempt, but fail, to ingest prey that are too large for them to swallow. To our knowledge, no study to date has investigated the influence of both prey and predator sizes on the frequency of ingestion success in a natural system.

The island of Guam (indigenous CHamoru name: Guåhan), in the tropical Western Pacific, offers a unique opportunity to conduct a detailed field study on the ingestion success of snakes. Brown Treesnakes (*Boiga irregularis*, BTS) became established on Guam after their accidental introduction following World War II, and subsequently spread across the island causing the local or total extinction of most native forest bird species (Savidge, 1987). BTS are a relatively large colubrid snake species, attaining a maximal length of over 3 m and weighing up to 200 times more as adults than as neonates (Jayne et al., 2022; Rodda et al., 1999). The diet of BTS on Guam is broad, including a variety of reptile, amphibian, bird, and mammal species, which are acquired through a mix of active and ambush foraging in arboreal and terrestrial habitats (Rodda, 1992; Rodda et al., 1999; Savidge, 1988). BTS have been linked with declines or extinctions in native prey species (Rodda & Fritts, 1992; Savidge, 1987), and indirect effects of their invasion are having ongoing and severe ecosystem‐wide repercussions (Rogers et al., 2017). While endothermic prey populations have collapsed in forested areas, commensal non‐native birds and mammals in urbanized areas continue to provide a relatively rich prey base for BTS (Savidge, 1991; Siers et al., 2017). Certain native bird species, including the omnivorous Såli (Micronesian Starling, *Aplonis opaca*), have also persisted and even recovered in urbanized areas, despite the ongoing threat of predation by BTS (Pollock et al., 2019, 2022; Wiles et al., 2003). Although all life stages of Såli are subject to BTS predation, fledgling birds experience particularly high mortality rates (Pollock et al., 2019).

In this study, we used radio‐telemetry to monitor the fate of fledglings from the largest remaining population of Såli on Guam. We captured BTS that successfully consumed Såli, and identified fledglings that were unsuccessfully ingested by BTS by their characteristic saliva‐coated head and nape (Pollock et al., 2019; Savidge, 1988). We also gathered data on the overall BTS population at the site by taking morphological measurements on all individuals found during visual surveys. We focused our study on four major questions: (1) what proportion of ingestion attempts by BTS on Såli fledglings is unsuccessful, (2) does the size of the prey predict ingestion success, (3) does the size of predator predict ingestion success, and (4) for successful ingestion attempts, what is the relative size of predators with respect to that of their prey?

## 2 MATERIALS AND METHODS

### 2.1 Study site and species

#### 2.1.1 Study site

We conducted our study between November 2019 and October 2022 on Guam, the largest island in Micronesia and southernmost in the Mariana Islands archipelago. Guam experiences a tropical climate with a dry season from January to June and a wet season from July to December. We studied interactions between Såli and their major predator, BTS, on Andersen Air Force Base (AAFB), an 8100 ha military installation on the northern tip of the island. Specifically, we focused on the Såli breeding range that spans roughly 500 ha in the urbanized southeastern portion of AAFB. This area is characterized by residential, commercial, and administrative buildings within expansive areas of lawn and isolated ornamental trees, bordered by native limestone forest to the east and mixed introduced forest interspersed with residential areas to the south. BTS control, primarily using poison bait and traps, was ongoing over some portions of the site during our study.

#### 2.1.2 Prey species

Såli are medium‐sized passerine birds (75 g upon fledging, 85 g as adults) in the starling family (Sturnidae) endemic to the Mariana and Caroline Islands (Baker, 1951). Although Såli are abundant across their range, they are locally endangered on Guam as a result of BTS predation, with a remnant population of around 1500 individuals that largely breed and roost within our study site (Pollock et al., 2022). Såli are year‐round cavity nesters, and on Guam, they mostly breed in artificial nest structures such as streetlamp posts, metal typhoon shutters, and nest boxes (Savidge et al., 2022). While Såli nest sites on AAFB are generally relatively safe from predation, fledglings suffer exceptionally high mortality rates in the first weeks after they leave the nest, primarily due to BTS and cats (~75%–95%; Pollock et al., 2019; Savidge et al., 2022).

#### 2.1.3 Predator species

Brown Treesnakes (BTS) are nocturnal, primarily arboreal, rear‐fanged snakes, with a native range spanning northern Australia, New Guinea, and some Melanesian islands, and a non‐native range restricted to Guam and some of its offshore islands (Barnhart et al., 2022; Rodda et al., 1999). On Guam, BTS inhabit all terrestrial habitats, with large individuals particularly common in urbanized areas where prey are more abundant (Savidge, 1991; Siers et al., 2017). BTS on Guam smaller than 700 mm snout‐vent length (SVL) are classified as juveniles, and those larger than 1025 (females) or 1030 (males) mm SVL as mature, while individuals between those size classes are considered to be in a transitional phase (Siers et al., 2017).

Brown Treesnakes exhibit an ontogenetic dietary shift, whereby juveniles feed almost exclusively on small lizards, but their diet expands to include larger endothermic prey such as birds and mammals as they enter the transitional phase (Lardner et al., 2009; Savidge, 1988). The size at which this shift occurs is variable; BTS as small as 650 mm SVL have eaten birds in captivity (Savidge, 1988), and the smallest snake in the wild on Guam is said to have consumed avian prey that measured 717 mm SVL (S. Siers, USDA, written communication, 2023). BTS larger than 900 mm SVL are consistently attracted to endothermic prey (Rodda et al., 2007), and, in urbanized habitats, the proportion of birds and mammals in their diet expands rapidly while the proportion of lizards declines to zero (Savidge, 1988; Siers, 2015). Avian populations on Guam appear to be particularly sensitive to the presence of BTS larger than 900 mm SVL (Nafus et al., 2024).

### 2.2 Fledgling survival monitoring

The Såli fledglings in our study hatched from an array of predator‐proof nest boxes (*n =* 70) installed across urbanized areas of AAFB (Savidge et al., 2022). Nestlings were banded at 22–23 days post‐hatching with a size 2 or 3 U.S. Geological Survey (USGS) metal band and a unique combination of three Darvic color bands to allow individual identification. At the majority of nests (72%), we also attached a radio‐transmitter (BD‐2, battery life ~175 days, Holohil Systems Ltd., Carp, Ontario, Canada; or PowerTag, battery life ~46 days, Cellular Tracking Technologies, Rio Grande, New Jersey, USA) to one or more nestlings per brood with a modified leg‐loop harness (Rappole & Tipton, 1991). Both transmitter types had a pulse rate of one pulse per 3 seconds. To avoid any deleterious effects, we ensured that the combined mass of the bands, harness, and transmitter weighed less than 4% of the nestling's body mass (Barron et al., 2010).

We conducted survival checks on Såli fledglings using handheld radio‐telemetry receivers. Fledglings were checked daily for the first 15 days post‐fledging, when mortality is the highest (Pollock et al., 2019), then three times per week from 16 to 30 days post‐fledging, and at least once per week from 31 days post‐fledging and beyond, until the transmitter stopped functioning due to depleted batteries. We obtained visual resights of fledglings and confirmed their identity using their unique color‐band combinations. The transmitter signal can still be picked up when it is inside a snake, so we were able to capture snakes after they successfully consumed a tagged fledgling.

**FIGURE 1:**
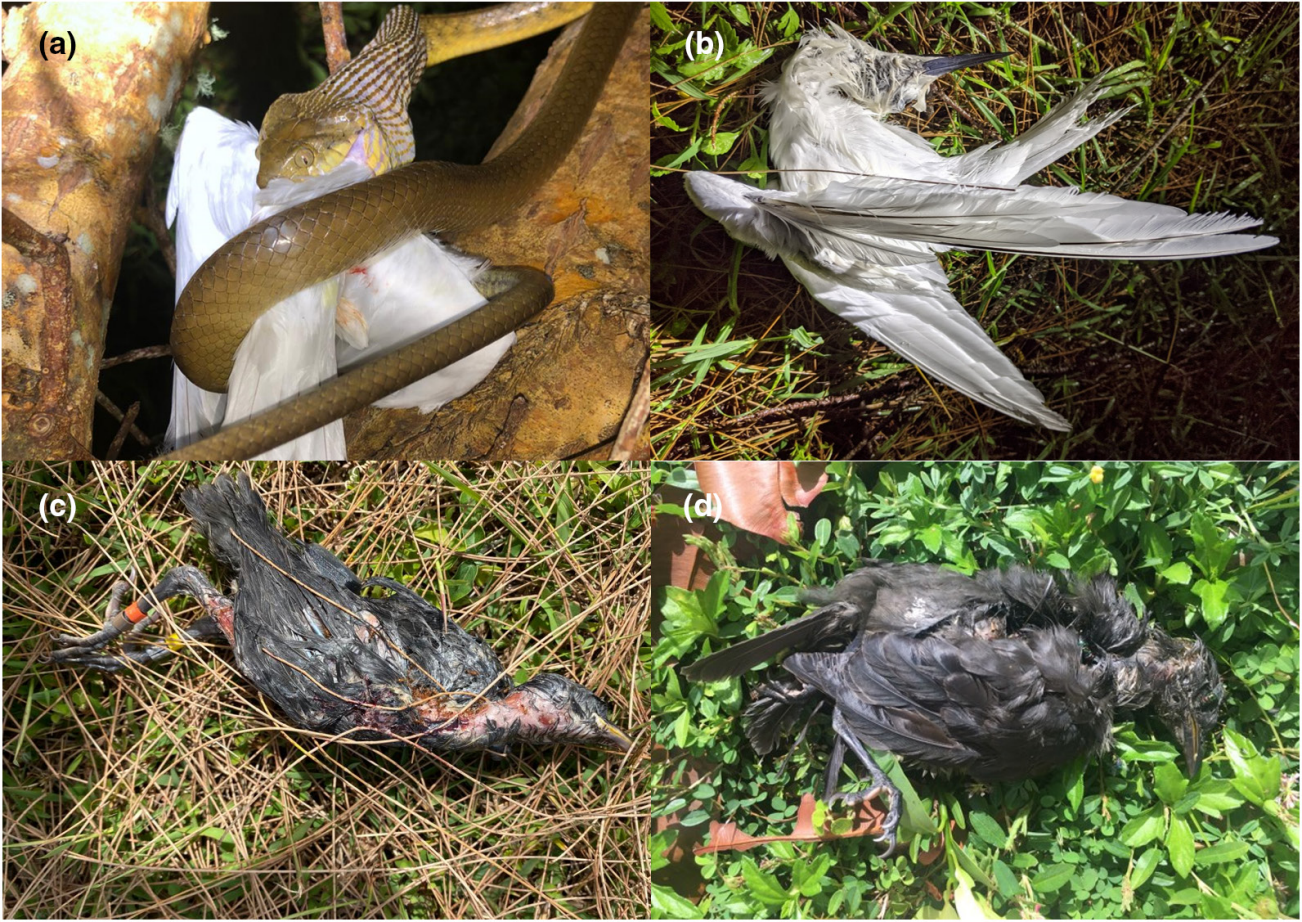
(a) Large Brown Treesnake (*Boiga irregularis*) in the process of ingesting a CHunge’ (White Tern, *Gygis candida*; photo credit: Nathan Sablan, public domain); (b) slimed (unsuccessfully ingested) adult CHunge’ found incidentally; (c) slimed fledgling Såli (Micronesian Starling, *Aplonis opaca*) found during telemetry work; and (d) slimed Såli adult found incidentally.

When we encountered a dead radio‐tagged fledgling, we assigned the cause of mortality as (1) successfully ingested by BTS if it was found inside a snake, or its transmitter had been gut‐passed by a snake (fecal matter found on the transmitter), (2) unsuccessful ingestion attempt by BTS (informally referred to as “slimed”) if the fledgling had characteristic saliva‐matted feathers on its head and body (Figure 1; Savidge, 1988; Pollock et al., 2019), (3) cat predation, if it had been partially or fully consumed with feathers, wings, and/or feet remaining, often accompanied by a broken transmitter harness, and (4) unknown, if it was found without signs of predation and without conclusive evidence indicating the cause of mortality. Other causes of mortality (e.g., starvation, entanglement) were assigned as they were encountered, based on available evidence. No predator species on Guam other than BTS would leave a saliva coating over birds' heads and bodies due to failed ingestion attempts or other handling.

When possible, we hand‐captured BTS that had consumed radio‐tagged fledglings during daily survival checks. When they were in inaccessible refugia (e.g., a small cavity), we returned nightly between four and six nights post‐consumption, when BTS re‐emerge following a period of reduced activity during digestion (Siers et al., 2018). Once captured, BTS were transferred to the Guam National Wildlife Refuge, where they were housed in a climate‐controlled room, in 5‐gallon buckets with water dishes and air vents, until they completed digestion and passed the Såli remains and transmitter. We then recorded morphometrics for the snake following the procedures described below, after which they were released at their point of capture. We were unable to capture the snake on 33 occasions, in which case we recovered the transmitter after gut passage to definitively confirm the fledgling's cause of death.

### 2.3 Brown Treesnake visual searches

We surveyed the BTS population at our study site between December 2019 and November 2022 to characterize the size distribution of snakes on the landscape. To do so, trained observers visually searched individual trees using high‐powered headlamps. We searched individual trees for a set amount of time that varied between 0:15 and 3:00 min depending on the structural complexity of the tree species. We included all snakes incidentally observed on the ground between directed searches of trees. We attempted to capture all observed snakes, and upon capture collected morphological data including SVL (mm), weight (g), and sex. Prior to release, we uniquely marked all snakes not previously captured with a subcutaneously injected passive integrated transponder and ventral scale clips, which allowed us to determine if the same snake successfully consumed multiple fledglings in our study.

Visual searching is the only known survey method to successfully sample all size classes of BTS (Rodda et al., 2007). However, results from a capture–mark–recapture study in forested habitat indicate that detection probabilities vary across BTS size classes, and visual searches may underrepresent the smallest and largest size classes (Christy et al., 2010). Because the smallest BTS size class is not attracted to endothermic prey (and thus irrelevant to our analysis), and large BTS are generally scarce, we do not believe that a bias toward midsized snakes is likely to substantively affect our results. Nevertheless, we caution that our resulting size distribution (Figure 3) should not be taken as a perfect reflection of relative BTS abundances across size classes on the landscape. Although relative detectability by size class across habitat types has yet to be assessed, we expect that overall detectability is likely to be higher in an open, urbanized setting such as our study site, compared to a more visually complex forested environment.

### 2.4 Statistical analyses

To quantify the proportion of Såli ingestion attempts by BTS that were unsuccessful, we calculated the proportion of Såli that were found slimed relative to those found ingested by BTS.

To assess whether the probability of being consumed by a BTS is related to prey size, we ran a generalized linear model with a binomial distribution with fledgling fate (consumed or slimed) as the dependent variable and fledgling mass as a continuous independent variable. We examined residual plots to assess model fit and used a Wald test to assess significance.

We used a bootstrapping approach to determine whether the size of the predator predicted ingestion success. While we could use the bird's transmitter signal to track and capture snakes that successfully ingested Såli fledglings, our methods did not allow us to locate snakes that failed to ingest birds; thus, we cannot directly compare the sizes of snakes that were successful in ingesting Såli to those that were not. However, we hypothesized that if snake size does not influence predation success, then the size of snakes captured after successful ingestion should not differ from a random sample of snakes on the landscape that are attracted to endothermic prey. Although smaller BTS have been recorded consuming avian and mammalian prey, we set the lower size threshold for the ontogenetic shift when BTS become consistently attracted to endothermic prey at 900 mm (Rodda et al., 2007). Therefore, we compared the size distribution of snakes captured after successfully consuming Såli to the size distribution of BTS larger than 900 mm SVL sampled in visual surveys across our study site. Because the sample of snakes that successfully consumed Såli was only 52, compared to a sample of 265 snakes that were captured through visual surveys, we repeatedly subsampled 52 snakes from the truncated visual survey dataset. We calculated the mean size for each of these 5000 samples, which gave us a distribution of mean sizes, from which we calculated 95% confidence intervals. We then determined whether the mean size of snakes found successfully ingesting Såli fell within the 95% confidence intervals of the distribution of mean sizes of endothermic‐prey‐eating snakes on the landscape.

We calculated relative prey mass for all successful predation attempts by dividing prey (Såli fledgling) mass by the mass of its respective predator (BTS). We used a linear model with a Gaussian distribution to test whether the mass of snakes (independent variable) is related to the mass of their prey (dependent variable). We visually inspected diagnostic plots of the residuals to assess model fit and used a Wald test to assess significance.

All analyses and graphing were performed using R Statistical Software (v4.2.2; R Core Team, 2022), including packages *boot* (Canty & Ripley, 2021; Davison & Hinkley, 1997) for the bootstrapping analysis, *ggResidpanel* (Goode & Rey, 2019) for examining model fit, and *ggplot2* (Wickham, 2016) for graphing. All values are presented as mean (μ) ± standard error (SE) unless otherwise stated, and we accepted significance at *α* < 0.05.

## 3 RESULTS

**FIGURE 2:**
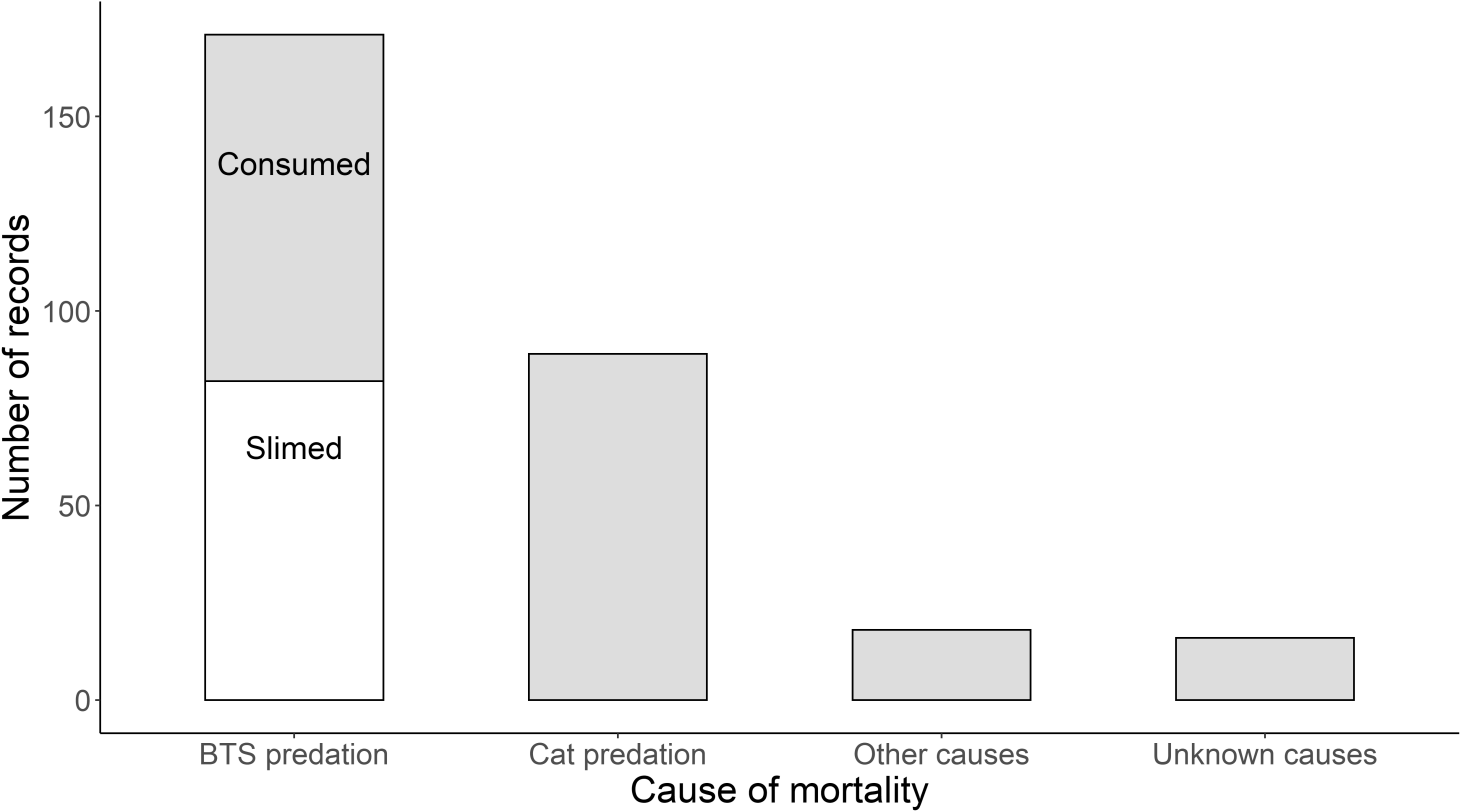
Records (*n* = 294 total) of Såli (Micronesian Starling, *Aplonis opaca*) fledgling mortalities from Andersen Air Force Base, Guam, monitored via radio‐telemetry between November 2019 and September 2022, by cause of mortality. BTS, Brown Treesnake (*Boiga irregularis*). Mortalities caused by unsuccessful ingestion by BTS (“slimed”, *n* = 82) are shaded white, all other categories are shaded gray. Mortalities identified as cat predation may represent direct kills or scavenging events. Other causes of mortality include starvation, exposure, road mortalities, and entanglement. The “unknown” category represents situations where it was not possible to determine the cause of mortality.

We recorded 294 Såli fledgling mortalities out of 461 fledglings tracked, of which 171 (58%) were attributed to BTS, and 89 (30%) to cats. Among BTS‐caused mortalities, 82 fledglings (48%) were slimed but not ingested by BTS and 89 (52%) were ingested (Figure 2). Two of the slimed fledglings had been scavenged by cats, but their mucus‐coated heads were present among the remains, allowing us to determine their primary cause of death. For the other mortalities attributed to cats, we were unable to differentiate between direct kills and scavenging events because there were insufficient carcass remains to allow a definitive determination.

The probability of being slimed or consumed by BTS did not depend on fledgling mass (*z* = −0.515, *p* = .890), which had a mean of 73.3 g ± 0.7, and a range of 60.5–86.3 g.

**FIGURE 3:**
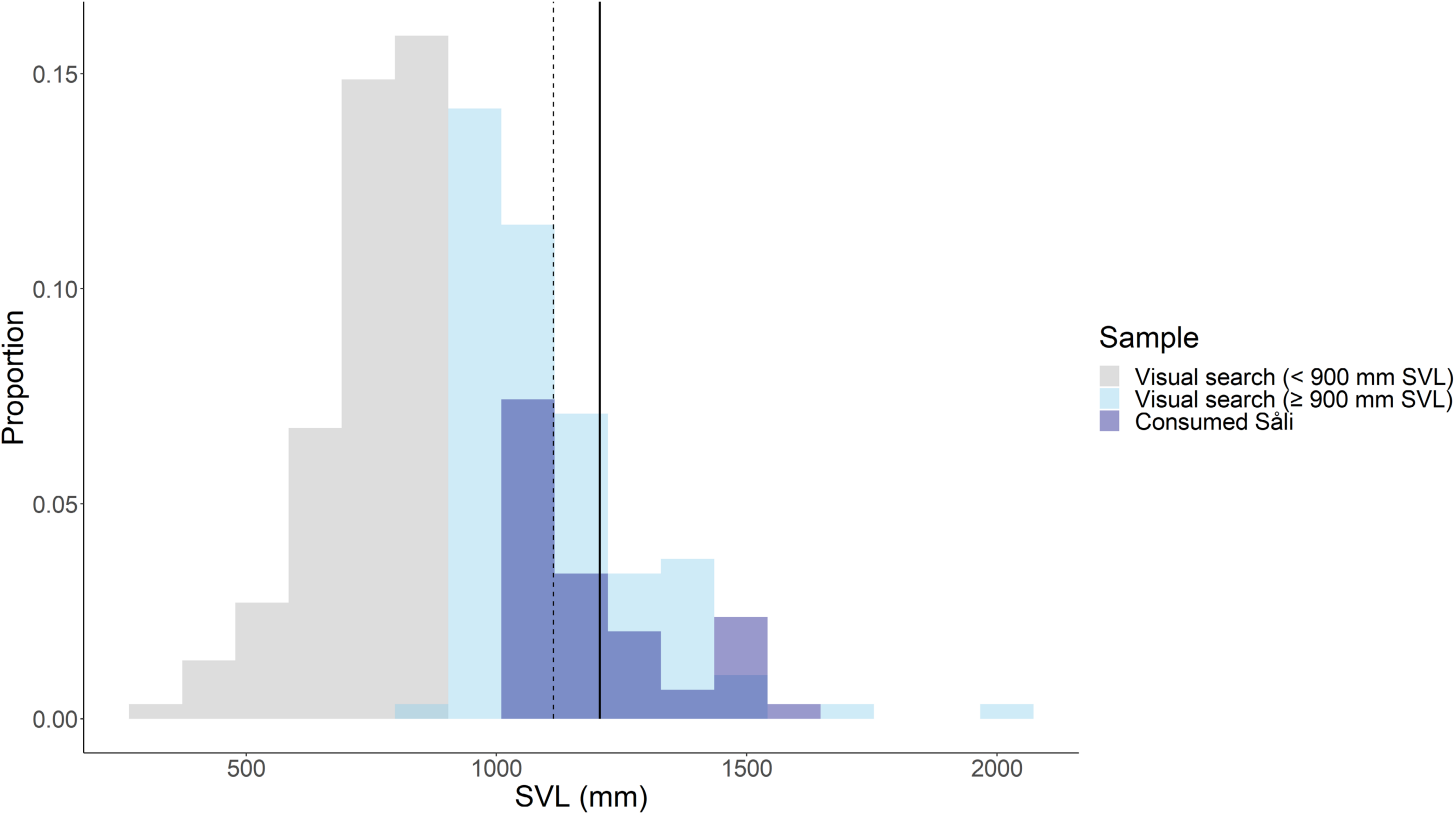
Histogram of snout‐vent length (SVL, mm) of BTS (Brown Treesnake, *Boiga irregularis*) found during visual searches across the study site (gray and light blue), and BTS that were captured after they successfully ingested Såli (Micronesian Starling, *Aplonis opaca*) fledglings (dark blue). The dotted vertical black line represents the bootstrapped mean (1114 mm SVL; 95% confidence intervals: 1068–1165 mm); the bold vertical black line represents the mean SVL of the sample of BTS that successfully ingested Såli (1206 mm). The threshold used to represent the minimum size of BTS attracted to endothermic prey was 900 mm SVL; BTS smaller than 900 mm SVL (gray bars) were excluded from the bootstrapping analysis. The y‐axis indicates the proportion that each class represents relative to all sampled snakes.

We captured 52 individual snakes that consumed Såli (55 total capture events, i.e., 3 individuals captured twice; 21 female, 28 male, and 3 of unknown sex), with a mean SVL of 1206 mm ± 23 (range: 1023–1593 mm). During visual surveys, we captured 265 individual snakes (354 total capture events, i.e., 89 recaptures; 131 were female, 121 male, 10 juvenile, and 3 of unknown sex), with a mean SVL of 933 mm ± 15 (range: 288–1989 mm). The mean SVL calculated from the means of each of our 5000 bootstrapped subsamples of 52 snakes was 1114 mm (1068–1165 mm 95% quantile means) for BTS over the 900‐mm threshold. The mean size of BTS found successfully ingesting Såli fell outside (above) the 95% confidence interval around the mean size of BTS larger than 900 mm SVL, indicating that snakes captured after successfully ingesting Såli were not a random sample of snakes on the landscape attracted to endothermic prey (Figure 3).

**FIGURE 4:**
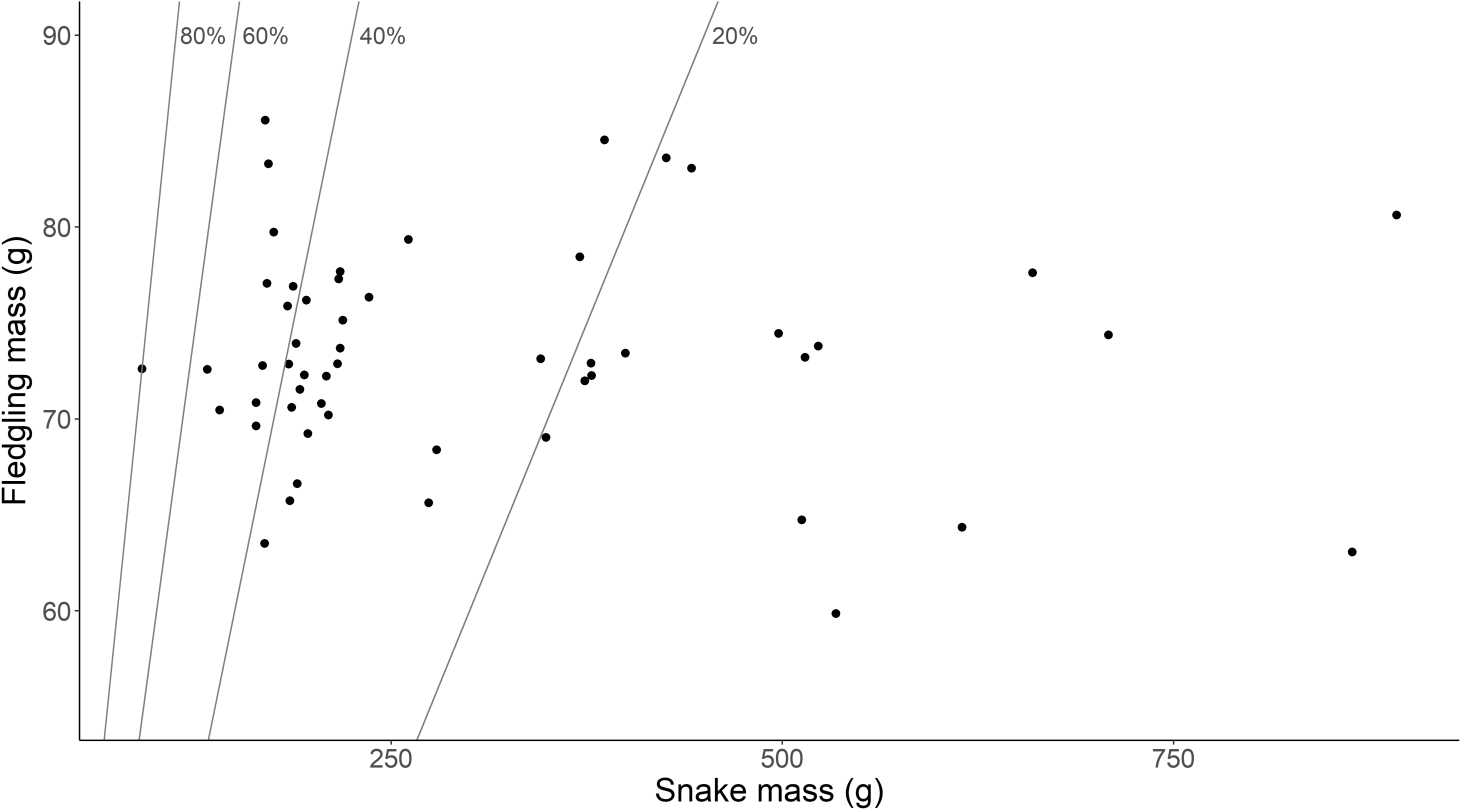
Scatterplot of BTS (Brown Treesnake, *Boiga irregularis*) mass (g) versus Såli (Micronesian Starling, *Aplonis opaca*) mass (g), whereby each point represents a relative prey mass (RPM = prey mass/predator mass) value for a BTS that successfully ingested a Såli fledgling. The gray lines represent the thresholds for RPM values of 20%, 40%, 60%, and 80%, and points to the right of those lines have RPM values below those respective thresholds. A point with RPM of 0.67 (67%) was excluded from this graphic because the snake ingested two fledglings in a single meal.

We did not find any relationship between snake mass and the mass of Såli fledglings they consumed (*t* = −0.627, *p* = .533). The mean relative prey mass (RPM) for snakes that successfully consumed Såli was almost one‐third of its own mass (31.5% ± 2.0). The smallest RPM value came from a prey item weighing only 7.2% of the mass of its predator, while the largest RPM value was found when a 90‐g snake successfully ingested a Såli fledgling that weighed 79.9% of its mass (71.9 g; Figure 4). In one exceptional case, a BTS consumed two Såli fledglings in one meal (resulting RPM = 67.2%), whereas in all other cases only a single fledgling was consumed per meal.

## 4 DISCUSSION

In this study, we documented that just under half of the radio‐transmittered birds predated by snakes were not successfully ingested. This figure is likely an underestimate because some of the mortalities attributed to cats may have initially been unsuccessful snake ingestion attempts, as we found cats to be scavengers at our study site. Furthermore, we found that while the size of prey in our sample does not predict the likelihood of successful ingestion by snakes, the size of the predator is predictive of successful ingestion. Specifically, snakes that successfully ingested Såli were significantly larger than snakes in the overall population that are attracted to endothermic prey. Finally, the smallest snakes that successfully ingested Såli attained very high relative prey mass values for their species (up to nearly 80%), indicating they may be approaching their “breaking point” (the threshold size for successful ingestion; Arnold, 1982). Although individual animals are known to occasionally consume prey that tax their anatomical limit for prey size, the frequency at which such events occur has remained an open question (Hertz et al., 1988; Jayne et al., 2022). Our results add to a growing body of evidence demonstrating that, in certain ecological contexts, snakes routinely attempt to consume prey items beyond the upper limit of their maximal gape (Robbins et al., 2019; Shine & Sun, 2003), with fatal effects on the prey and no documented impacts on the predator.

The relative prey mass figures we measured are the highest ever recorded for Brown Treesnakes (Chiszar, 1990; Rodda et al., 1999), and among the highest for any colubrid snake species (Glaudas et al., 2019). This is particularly remarkable because volant birds, such as Såli, have relatively low body densities compared to mammalian or reptilian prey (Jayne et al., 2022). Although our methods did not allow us to directly sample snakes that were unsuccessful in ingesting birds, our results strongly indicate that intermediate‐sized snakes, the most abundant size class on the landscape (Figure 3), are likely responsible for the bulk of the unsuccessful ingestion events we observed. This inference aligns with the observations of Shine and Sun (2003) that small *Gloydius shedaoensis* regularly fail to swallow excessively large prey due to gape limitation. Our results therefore support the prediction of Feder and Arnold (1982) that snakes would routinely attack large prey items even if they are ingested only occasionally, at least in instances where subduing the prey entails limited risk of injury and low energetic costs. However, the results contradict the prediction that predators behaving optimally would avoid attacking prey larger than their gape limit (Christensen, 1996), although the extent to which predators adopt optimal foraging strategies outside of their native ranges remains an open question.

Brown Treesnakes (BTS) on Guam were previously thought to predate all life stages of smaller bird species, but only eggs and nestlings of larger species, because they would be incapable of swallowing larger prey (McElderry et al., 2021; Savidge, 1987). Wiles et al. (2003) found a negative relationship between body size and date of extirpation in forest birds on Guam, with smaller birds disappearing first, but no such relationship when the sample was extended to include birds from other habitats also affected by BTS, such as resident seabirds. It may be that behavioral adaptations among some of Guam's larger species (e.g., communal roosting in Mariana crow *Corvus kubaryi*, Wiles, 1998), rather than reduced snake predation on larger birds, drove the relationship between size and persistence in the forest bird sample. We did not detect any relationship between body size of Såli fledglings and BTS ingestion success in our study. However, our sample was restricted to a single species with a relatively narrow body mass range (60–86 g). We might expect a negative relationship between size and probability of ingestion to emerge over a wider range of prey sizes. Moreover, it is probable that snakes will generally not try to feed on prey that vastly exceed their gape limit, even if such attempts are known to occur occasionally (e.g., Fritts et al., 1994; Natusch et al., 2021). For example, Shine and Sun (2003) found that small *Gloydius shedaoensis* regularly made feeding strikes at decoys too large for them to conceivably ingest, but that an upper threshold for eliciting strikes did exist (i.e., small snakes did not strike at the largest decoys). Therefore although the largest prey species on Guam may have reduced risk of predation, the willingness of BTS to attack prey items too large for them to swallow may preclude a size refuge for most native bird species. One strategy for recovering avian populations on Guam has proposed focusing control efforts on the removal of the largest snakes on the landscape, in order to remove those individuals capable of consuming birds (e.g., Klug et al., 2021). However, our study demonstrates that snake management for bird restoration would need to account for fatal predation attempts by snakes too small to successfully ingest their prey. This is particularly important in light of the results of McElderry et al. (2022), which indicate that reintroduced native bird populations on Guam will have extremely low predation thresholds to allow population establishment.

Our results favor the suggestion that the benefit to predators of attempting to consume large prey items may outweigh the inherent risk of such behavior if the potential energetic gain is substantial (Arnold, 1993; Natusch et al., 2021). The risk of asphyxiation may be considerable for snakes that attempt to consume oversized elongated (but not gape‐limiting) prey, such as other snakes or amphisbaenians (e.g., Caramaschi & de Niemeyer, 2012), which would cause unremittent pressure on the lung for the entire consumption attempt. Indeed, Collins and Rodda (1994) found that feeding BTS mice that were stitched together end‐to‐end (weighing 52% of the snake's body mass) caused near‐universal regurgitation and two fatal cases of asphyxiation in a sample of 12 snakes. However, despite documenting over 170 BTS ingestion attempts, with most of the successful attempts attaining high relative prey mass values (>30%), we did not encounter any BTS that died while attempting to ingest Såli. Two BTS (one of which had consumed two Såli fledglings) died while regurgitating their meals in captivity; however, the regurgitation may have been triggered by handling stress (Crum, 2012). It appears that predation attempts on gape‐limiting prey, regardless of whether ingestion occurs, may pose a relatively low risk to snakes, assuming the prey can be safely incapacitated.

If the energetic gain from successful ingestion of large prey outweighs the cost of unsuccessful attempts, then conditions may favor attempts where failure is a possible outcome. Feder and Arnold (1982) estimated that the metabolic cost of predatory activity to Terrestrial Garter Snakes (*Thamnophis elegans*) was less than 1% of the energy assimilated from the prey. Cruz‐Neto et al. (1999) tested the aerobic metabolism of juvenile South American Rattlesnakes (*Crotalus durissus*) ingesting prey ranging from 10% to 50% of their body mass, and found that the energy needed for prey ingestion represented only 0.02% of the energy content of the largest prey, and that aerobic metabolism during ingestion was relatively more efficient for larger prey than for smaller prey. Similarly, Canjani et al. (2002) found that the energy spent by *Boa constrictor* to constrict, inspect, and ingest prey ranging from 5% to 40% of their body mass ranged from 0.21% to 0.11% of the energy assimilated from the prey, respectively. The energetic costs of prey handling and ingestion for snakes appear to be trivial compared to the potential rewards, and consumption of larger prey may be more efficient than consumption of smaller prey. The most energetically costly aspect of feeding by snakes is the digestive process, which can involve major upregulation of gut function, particularly for species that feed infrequently (Jackson & Perry, 2000; Secor, 2008). Although evidence in this regard is mixed, it has been hypothesized that the major energetic investment involved in digestion may be another incentive for snakes to maximize the size of their prey (Glaudas et al., 2019; Secor & Diamond, 1998; Shine & Sun, 2003).

Evolutionary theory predicts that a high rate of unsuccessful predation attempts should be expected if attempts are cheap and prey is valuable (Abrams, 1989). The work of others demonstrates that ingestion attempts for snakes are cheap and that large prey is valuable, while our results confirm that unsuccessful ingestion of birds may be more prevalent, and less risky, than previously recognized. Certain aspects of Guam's ecology, such as a substantial size gap between reptilian and most endothermic prey (Savidge, 1988), may promote ingestion attempts by snakes on oversized prey. However, the fact that this phenomenon was previously unrecognized despite several decades of intensive research on BTS, alongside comparable results from other systems where the predators and prey have coevolved (Robbins et al., 2019; Shine & Sun, 2003), indicated that high rates of unsuccessful ingestion by snakes can easily remain undetected without dedicated monitoring of prey species. Indeed, the large proportion of unsuccessful ingestion attempts we recorded would not have been captured by traditional techniques such as dissection of museum specimens or regurgitation by palpation, highlighting the utility of alternative methodologies in acquiring a more complete picture of the outcomes of predation attempts (Glaudas et al., 2017). Intensive monitoring of both predator and prey species is crucial to gain a deeper understanding of the complexities of predator–prey interactions. Additional research in other systems would help to uncover the full scale of unsuccessful ingestion by predators and its importance in shaping ecological dynamics.

## AUTHOR CONTRIBUTIONS

**Martin Kastner:** Conceptualization (equal); data curation (equal); formal analysis (equal); investigation (equal); methodology (equal); visualization (lead); writing – original draft (lead); writing – review and editing (lead). **Scott M. Goetz:** Conceptualization (equal); data curation (equal); investigation (equal); methodology (equal); writing – review and editing (supporting). **Kayla M. Baker:** Investigation (equal); writing – review and editing (supporting). **Shane R. Siers:** Writing – review and editing (supporting). **Eben H. Paxton:** Writing – review and editing (supporting). **Melia G. Nafus:** Conceptualization (equal); funding acquisition (equal); methodology (equal); project administration (equal); writing – review and editing (supporting). **Haldre S. Rogers:** Conceptualization (equal); formal analysis (equal); funding acquisition (equal); methodology (equal); project administration (equal); writing – review and editing (supporting).

## FUNDING INFORMATION

This research was supported by the Department of the Navy, Joint Region Marianas (the “Navy”), through cooperative agreements (N40192‐19‐2‐8006 and N40192‐22‐2‐8000) between the Navy and Iowa State University; the United States Geological Survey (USGS), and Military Interdepartmental Purchase Requests M2002118MPDP007 and N6175519GTC3396 with the USGS.

## CONFLICT OF INTEREST STATEMENT

The authors declare no conflict of interest.

### OPEN RESEARCH BADGES

This article has earned an Open Data badge for making publicly available the digitally‐shareable data necessary to reproduce the reported results. The data is available at https://github.com/EBL‐Marianas/Sliming; https://doi.org/10.5066/P14Q2JCZ.

## Data Availability

Virginia Tech data and code for this study are available at https://github.com/EBL‐Marianas/Sliming. United States Geological Survey data are available at https://doi.org/10.5066/P14Q2JCZ (Goetz & Nafus, 2024).
